# Molecular Mechanisms of Treadmill Therapy on Neuromuscular Atrophy Induced via Botulinum Toxin A

**DOI:** 10.1155/2013/593271

**Published:** 2013-11-12

**Authors:** Sen-Wei Tsai, Hsiao-Ling Chen, Yi-Chun Chang, Chuan-Mu Chen

**Affiliations:** ^1^Department of Life Sciences, Agricultural Biotechnology Center, National Chung Hsing University, Taichung 402, Taiwan; ^2^Department of Physical Medicine and Rehabilitation, School of Medicine, Tzu Chi University, Hualien 970, Taiwan; ^3^Department of Physical Medicine and Rehabilitation, Taichung Tzu Chi Hospital, Buddhist Tzu Chi Medical Foundation, Taichung 404, Taiwan; ^4^Center of General Education, National Taichung University of Science and Technology, Taichung 404, Taiwan; ^5^Department of Bioresources, Da-Yeh University, Changhua 515, Taiwan; ^6^Rong Hsing Research Center for Translational Medicine, National Chung Hsing University, Taichung 402, Taiwan; ^7^Integrative Evolutionary Galliform Genomics (iEGG) Center, National Chung Hsing University, Kuo Kuang Road, Taichung 402, Taiwan

## Abstract

Botulinum toxin A (BoNT-A) is a bacterial zinc-dependent endopeptidase that acts specifically on neuromuscular junctions. BoNT-A blocks the release of acetylcholine, thereby decreasing the ability of a spastic muscle to generate forceful contraction, which results in a temporal local weakness and the atrophy of targeted muscles. BoNT-A-induced temporal muscle weakness has been used to manage skeletal muscle spasticity, such as poststroke spasticity, cerebral palsy, and cervical dystonia. However, the combined effect of treadmill exercise and BoNT-A treatment is not well understood. We previously demonstrated that for rats, following BoNT-A injection in the gastrocnemius muscle, treadmill running improved the recovery of the sciatic functional index (SFI), muscle contraction strength, and compound muscle action potential (CMAP) amplitude and area. Treadmill training had no influence on gastrocnemius mass that received BoNT-A injection, but it improved the maximal contraction force of the gastrocnemius, and upregulation of GAP-43, IGF-1, Myo-D, Myf-5, myogenin, and acetylcholine receptor (AChR) subunits **α** and **β** was found following treadmill training. Taken together, these results suggest that the upregulation of genes associated with neurite and AChR regeneration following treadmill training may contribute to enhanced gastrocnemius strength recovery following BoNT-A injection.

## 1. Introduction

Treadmill exercise, both full weight-bearing and partial weight-bearing, is a dynamic training approach that provides intervention for walking and gait analysis. In patients with neuromuscular disorders, such as stroke, spinal cord injury (SCI), or cerebral palsy (CP), treadmill exercise is a frequently used rehabilitation training model that has been shown to yield functional improvements [1–4]. Clinical investigations showed that in patients with CP, treadmill training can improve walking endurance, walking speed, and standing performance [5, 6]. In stroke rehabilitation, partial-support treadmill training is also a widely used training mode for gait correction [7, 8]. Spasticity is a sign of upper motor neuron lesion with increased stretch reflex depending on movement velocity, which can be caused by stroke, spinal cord injury, brain injury, cerebral palsy, or other neurological conditions [9]. One of the treatment choices for spasticity is the intramuscular injection of botulinum toxin A (BoNT-A) [10, 11]. Although several studies support the beneficial effects of treadmill training, most excluded BoNT-A-treated patients or did not mention these patients [12–14]. The effects of treadmill training on the physiological adaptation to paralysis effects caused by BoNT-A remain poorly understood. In this paper, we review the mechanisms of treadmill exercise and BoNT-A treatment and discuss their combined effects on the central nervous system, physiological activity, and changes in the muscle and neuromuscular junction (NMJ). This may contribute to our understanding of the mechanisms underlying currently used treatments and, possibly, suggest directions for future research.

## 2. The Therapeutic Effects of Treadmill Training and Mechanism

In neurorehabilitation, locomotor training is based essentially on principles that promote the movement of the limbs and trunk to generate sensory information consistent with locomotion. Whether full weight-bearing or partial-weight bearing, treadmill training can be used as a strategy for locomotor training in people with certain disabilities to improve muscle adaptation and walking ability. A major focus of research has been to elucidate the benefits of treadmill training, such as functional recovery or restoration in neural plasticity. One of the major questions limiting the rehabilitative implementation of treadmill training pertains to the molecular mechanisms through which treadmill training promotes synaptic plasticity and functional recovery. Clinical investigations have shown beneficial effects of treadmill training, which is often used in patients with cerebral palsy (CP) or stroke for walking and gait training [13–16]. In patients with CP, walking speed and gross motor function improved significantly after treadmill training [17]. A recent systemic review showed that gait impairment and activity level were improved after body weight supported treadmill training [16]. Recently, robotic-assisted treadmill training was developed and was found to improve walking and standing performance in patients with CP [18]. In patients with CP, the neural modulation of soleus H-reflex suppression was proposed as the mechanism accounting for the improvement in functional gait pattern after treadmill training therapy [19].

In animal models of SCI, locomotor training using a body weight supported treadmill (BWST) suggested that interneurons in the lumbar cord formed circuits for rhythmic and alternating hindlimb flexion-extension movement [20, 21]. Because this conceptual mechanism included the responsiveness of the spinal central pattern generators to sensory input with locomotion, BWST training provides an environment in which one can learn to execute the stepping leg movement [22–24]. The amplitude and coordination of the firing of motor units in leg muscles were also found to increase after considerable BWST training in patients with complete or incomplete chronic SCI. The animal and human studies led to the suggestion that BWST training may tap into this central pattern generator subsystem and contribute to enabling walking in highly impaired patients [25–28]. Treadmill training also increased the expression of nerve-associated factors, such as the brain-derived neurotrophic factor (BDNF) and neurotrophin-3 (NT-3) in the spinal cord; this expression may be related to the improvement in local neural circuitry [29–33]. Although an isolated spinal cord learned to stand on a stationary treadmill or step on a moving treadmill [34], the training effect for SCI did not transfer to the other task [35]. Hence, the cord has a limited capacity for relearning multiple tasks in the absence of supraspinal input [36]. Thus, factors such as task specificity, training intensity, or training duration are issues that warrant attention in future experiments [37]. 

Although injured axons in peripheral nerves have better regeneration than those in the central nervous system and despite the recent advances in microsurgical techniques, the functional outcomes in injured peripheral nerves are clinically poor [38–40]. Some studies had evaluated the effects of treadmill training on axon regeneration following peripheral nerve injury. In animal studies of nerve transection following repair, treadmill training was shown to facilitate growth in the length of regenerating axons, to retrieve restoration of H-reflex, and to increase the amplitude of CMAP in injured peripheral nerves [41, 42]. In the sciatic nerve crush animal model, Ilha et al. [43] found improvement in sciatic functional index (SFI) scores and a better morphology of regenerating nerve fibers after treadmill training. As BDNF is highly expressed in active neurons, BDNF-mediated machinery may be responsible for the spinal central pattern generation induced using treadmill locomotor training [44–46]. Both the Wilhelm group [47] and Ying and colleagues [30] provided evidence that the effect of treadmill training on axon regeneration requires BDNF produced by the regenerating axons themselves. This neurotrophic factor was a likely mechanism of the effect of treadmill training in enhancing axon regeneration following peripheral nerve injury. 

In normal rats, the adaptation of the energy transportation system was found after treadmill training. Chow and colleagues demonstrated that after 8 weeks of training, a significant increase in mitochondrial-related mRNAs was observed [48]. They also found that mitochondrial DNA and mitochondrial transcription factor A were upregulated in the trained muscle. Safdar and colleagues have advocated treadmill endurance exercise as a medicine and a lifestyle approach to improve systemic mitochondrial function. They showed that 5 months of exercise resulted in a substantial increase in mitochondrial oxidative capacity and respiratory chain assembly, restored mitochondria morphology, blunted the process of apoptosis, and prevented mitochondrial DNA depletion and mutations [49].

Several muscle adaptation mechanisms have been observed in normal or diseased animal models following treadmill training. In one recent study that measured changes in denervated soleus muscle via sciatic nerve resection and treadmill training, Jakubiec-Puka et al. [50] showed that the number of capillary blood vessels, amount of myosin heavy chains, and muscle fiber nuclei were increased, with concomitant decreases in the number of severely damaged muscle fibers and amounts of collagen. These training effects were more evident in the animals with longer training [50]. In diabetic rats, treadmill running has been shown to increase the level of nerve growth factor in the soleus muscle, and apoptotic cell death was suppressed via accelerating p-PI3-K activation [51]. In summary, the adaptation mechanisms induced via treadmill exercise are multifactorial with cellular changes inside the muscle fibers as well as changes in peripheral and central nervous systems. 

## 3. Efficacy and Reliability of Current Measures of Spasticity 

Spasticity is a clinical symptom of upper motor neuron lesion that is characterized by a velocity-dependent increase in stretch reflexes [9]. Although some objective methods of measuring spasticity such as the Hoffmann reflex (H-reflex), the Tendon reflex (T-reflex), and the Stretch Reflex (SR) have been developed, the clinical and experimental use of the three methods is limited due to moderate reliability and sensitivity [52]. Clinically, the six-point-ordinal Modified Ashworth Scale (MAS) is now the most commonly used measure of spasticity [53]. Mutlu et al. showed that in cerebral palsy, the MAS is a marginally reliable assessment of spasticity. They suggested that the use of the scale should therefore be interpreted with great caution [54]. In another study evaluating the reliability for ankle plantar flexor in patients with traumatic brain injury, a low reliability was concluded [55]. Although some controversy to the MAS approach has been recognized, most of the literature supports the reliability of the MAS. Ghotbi et al. [53] showed that the reliability was good for the distal ankle plantar flexors but not for the proximal hip adductors. Bohannon and Smith [56] have advocated the MAS as a reliable test of elbow flexor muscle spasticity. In an assessment of knee extensor, Ansari and colleagues [57] showed a good reliability for MAS evaluation on the poststroke knee extensor. Pandyan and colleagues [58] showed that the reliability of the scale is better in the upper limb. Platz et al. [59] suggested that a high interrater reliability of the MAS can be clinically achieved but not in all circumstances. Therefore, we contend that the clinical reliability of applying the MAS for spasticity evaluation may depend on the joints and muscles tested [54, 60]. 

## 4. Neuromuscular Junction: Structure and Molecular Mechanism

The neuromuscular junction (NMJ) in vertebrates is a favorable model system for investigating the molecular mechanisms of synapse formation and neural plasticity. The NMJ is a region where the axons of motor nerves connect with the skeletal muscles and serves to efficiently communicate the electrical impulse from the motor neuron to the skeletal muscle to signal contraction [61, 62]. Neurotransmitters, such as acetylcholine (ACh), are formed in the neuron body and then transported to the synapse along the axon. In the terminal axon of a nerve, neurotransmitters are packed in vesicles. When an impulse from the central nervous system is transmitted to the NMJ, ACh is released, which binds with acetylcholine receptors on the postsynaptic muscle fibers [63]. Calcium-related signal transduction will be recruited, causing muscle contraction. 

The nicotinic acetylcholine receptor (AChR) is a transmembrane ligand-gated ion channel. This receptor is composed of four homologous subunits: *α*, *β*, *δ*, and *γ* or *ε* [64]. During myogenesis, the expression of the muscle regulatory factors (MRFs) family is associated not only with activated satellite cells and myonuclei but is also crucial in regulating the ongoing rates of AChR gene transcription [65–67]. The expression of the AChR subunits and the distribution of these receptors among muscular fibers are regulated developmentally, with AChR gene expression at its highest levels during myogenic differentiation [68]. The soluble N-ethylmaleimide-sensitive-factor attachment protein receptor (SNARE) is the most widely studied element of the intracellular machinery involved in intracellular trafficking [69]. SNARE proteins are a large protein superfamily consisting of more than 60 members. The core exocytotic machinery is composed of three SNAREs: (1) vesicle-associated membrane protein synaptobrevin (VAMP), (2) synaptosomal-associated protein of 25 kD (SNAP-25), and (3) syntaxin-1 on the plasma membrane [70–73].

## 5. Botulinum Toxin A (BoNT-A): Structure and Cellular Mechanism

The botulinum toxin was first described as a “sausage poison” and “fatty poison” because this bacterium often caused toxicity by growing in improperly handled or prepared meat products [74]. In the late 1960s, Scott and Schantz were the first to work on a standardized botulinum toxin preparation for therapeutic purposes. Scott, an ophthalmologist, first applied tiny doses of the toxin to treat “crossed eyes” (strabismus) and “uncontrollable blinking” (blepharospasm) [75]. In December 1989, BoNT-A (Botox, Allergan Inc., Irvine, CA, USA) was approved by the US Food and Drug Administration (FDA) for the treatment of strabismus, blepharospasm, and hemifacial spasm in patients over 12 years old. Dysport (Ipsen Ltd., UK) is another brand of BoNT-A used for therapeutic purposes. 

In the peripheral and central nervous systems, neuronal plasticity plays a pivotal role in the recovery process after injury. However, the intrinsic neuronal determinants for the regulation of this fundamental process remain poorly defined. The intramuscular injection of botulinum toxin is a unique strategy for investigating the process of neuronal plasticity in motor nerves and entails the elimination of regulated neurotransmitters while leaving the viability of the nerve endings unaltered [76]. Seven botulinum neurotoxins (A to G) have been found, and all act in the postsynaptic cholinergic nerve terminals [10]. BoNT-A is a type of bacterial zinc-dependent endopeptidase that acts specifically at the neuromuscular junction [10, 77]. The complex of BoNT-A comprises a 150 kD neurotoxin protein, as well as nontoxin nonhemagglutinin proteins. The 150 kD neurotoxin protein is the biologically active component, while the nontoxin nonhemagglutinin protein stabilizes and protects the active neurotoxin component [78]. The 150 kD neurotoxin protein is pharmacologically inactive until the disulfide bond is cleaved to form one 100 kD heavy chain and one 50 kD light chain.

After the endocytotic uptake of BoNT-A from postsynaptic terminals, the light chain of BoNT-A cleaves SNAP-25 [79, 80]. This renders ACh-containing vesicles unable to dock and fuse to the presynaptic membrane. By inhibiting the release of acetylcholine at the NMJ, neuroparalysis and denervation of the involved muscles occur, which decreases the ability of the muscles to generate force [79, 80]. Once paralysis has been produced by BoNT-A, new nerve sprouting is elicited, and the newly created synapses are responsible for the initial synaptic transmission [81]. A previous study showed that the effect of botulinum toxin lasts for approximately 3 to 6 months. The muscle that received the BoNT-A injection then regains muscle mass and recovers its contraction ability [82]. 

## 6. Changes in Muscle Physiology, Neuromuscular Junction, and Gene Expression following BoNT-A Injection

One to two weeks after BoNT-A injection, muscle mass and force were significantly reduced but returned to nearly normal at 3–6 months after injection. Studies showed that muscle mass following BoNT-A injection was reduced by approximately 70% to 30% within 1 to 6 months [82–84]. A 30% to 90% (approximately) reduction in muscle force was reported in animal studies [83, 85–87]. The wide range of reduction in muscle mass and force generation occurred in a dose-dependent manner [85, 88]. 

The mass and structural integrity of contralateral muscles that received BoNT-A injection and those of noninjected peripheral muscles were affected in both clinical and animal studies. Clinically, the diffusion of the injected BoNT-A to adjacent muscles was reported in patients with spasmodic torticollis, facial hemispasm, blepharospasm, or palmar hyperhidrosis [89–91]. Fortuna et al. [83] showed that muscle atrophy and decreased muscle force were observed in the quadriceps muscles of the contralateral hindlimbs. In a rat model that used the contralateral gastrocnemius muscle for comparison, the injected toxin was found to have no effect on the force of the contralateral leg using a 1 unit/kg injection dose. This toxin spreading effect was suggested to be dependent on toxin dosage [92]. 

Neuroparalysis produced via BoNT-A elicits nerve sprouting and newly created synapses that are responsible for the initial synaptic transmission at the onset of recovery [81, 93]. However, whether synaptic transmission occurs at the newly developed sprouts has not been directly demonstrated. Recently, Rogozhin and colleagues [94] advocated that the original synaptic sites play the predominant role in functional restoration after BoNT-A rather than the nerve sprouts, as previously thought. At approximately 90 days after exposure to BoNT-A, the restoration of parent NMJ functioning and a concomitant retraction of the outgrowth neurites could be found [76]. 

Following BoNT-A injection, genes related to NMJ remodeling and myogenesis, including subunits of AChR, IGF-1, MRFs, MuSK, and p21, eventually lead to NMJ stabilization and muscle function recovery [95–97]. In neuromuscular disorders, an electrophysiological study is an objective evaluation tool. A treadmill walking study in cats showed that after a temporal reduction in ankle extensor activity via BoNT-A injection, the functional deficit recovery was not associated with the return of the electromyogram (EMG) pattern [98]. However, the EMG burst of the synergistic muscle that was not poisoned by BoNT-A was increased. The authors concluded that this early functional recovery is not due to muscle hypertrophy but is instead attributable to neuronal adaptation due to an increased gain in stretch reflex or central drive [99, 100]. The compound muscle action potential (CMAP) represents the summation of a group of nearly simultaneously activated action potentials from a muscle or a group of muscles that are innervated by the same nerve. The reduction in CMAP parallels the decrease in the mean rectified voltage during a maximal voluntary contraction [101]. A recent study showed that the CMAP amplitude was significantly reduced, while no changes of distal latency were found in the gastrocnemius following a BoNT-A injection for 4 weeks [84]. This result was compatible with the results of a previous study demonstrating that an injection of BoNT-A caused localized muscle paralysis but no disruption in axonal transport [102]. After BoNT-A injection, the CMAP amplitude typically requires more than 3 months for full recovery [101].

## 7. Treadmill Training Models and the Training Effects on Muscle Activity and NMJ following BoNT-A Injection

In rat exercise training models, the two most frequently used models are either voluntary wheel running or forced treadmill training. In the wheel running training model, voluntary running activity occurs in a nonstressed environment. However, the training speed and duration are technically challenging to monitor [103–105]. The alternative to wheel running is treadmill training. In the treadmill training model, different training paradigms have been used. Some groups use ramp protocols [106], and others use the model with a consistent speed and exercise duration [84, 107]. The advantage of treadmill training is that the animals can be made to exercise at a desirable training intensity and duration. However, the experimental conditions are often stressful, and the training pattern is far removed from normal mouse behavior [108]. 

The interaction effect between treadmill and BoNT-A injection has not been clearly demonstrated. Combined BoNT-A injection and 7 days of voluntary wheel running exercise in juvenile rats attenuated the BoNT-A-induced loss in muscle fiber size [109]. Although the number of Myo-D positive nuclei was increased after BoNT-A injection, the results showed that exercise had no effects on myonuclear production. The authors concluded that this early effect of combined BoNT-A and exercise may be due to the passive stretching of paralyzed muscle fibers. This passive stretch effect was supported by a study that showed an increased expression of mechanosensitive CARP and the Ankrd2 gene in rats receiving BoNT-A gastrocnemius injection and 3 weeks of wheel running exercise [110]. 

In a study by Chen et al. [109], a muscle atrophy attenuation effect was observed in gastrocnemius following BoNT-A injection after 1 week of exercise. Another study found that muscle mass was not changed after 3 weeks of wheel running. Recently, Tsai et al. showed that after BoNT-A injection, the gastrocnemius mass did not increase after 4 weeks or 8 weeks of treadmill training [84, 86]. As previous studies have shown that there is no significant effect of treadmill training on muscle mass following BoNT-A injection, it is reasonable to postulate that the strength of muscle following BoNT-A with or without treadmill training is likely unchanged [84, 86, 110]. However, one recent study showed that after the intramuscular injection of BoNT-A into the gastrocnemius, treadmill training improved the recovery of muscle contraction strength [85]. In the study, an increase in CMAP amplitude was observed in the gastrocnemius of BoNT-A-injured rats after 4 weeks of treadmill running. This functional improvement was confirmed via the improvement of the sciatic functional index (SFI). In sciatic nerve injury, rats lose their ability to spread their hind toes. The SFI is an experimental method used for the functional assessment of the extent of sciatic nerve injury and for the monitoring of recovery [111–113]. In one recent study, Tsai et al. [87] demonstrated an increase in IGF-1, GAP-43, MyoD, Myf-5, and myogenin expression, as well as the upregulation of AChR *α* and -*β* subunit expression, in the BoNT-A-paralyzed gastrocnemius after 8 weeks of treadmill running. Synaptic transmission at the NMJ is mediated through the AChR, and control of AChR transcription is crucial for the regeneration and maintenance of synapses in muscle. The expression and transcription of AChR genes are governed by the sequential expression of MRFs [65–67]. Charbonnier et al. [66] showed that when the neuromuscular junction begins to differentiate, MyoD, Myf-5, and MRF4 display different specificities for the transactivation of the genes encoding the different subunits of the AChR. Taken together, after treadmill training the upregulation of IGF-1, GAP-43, MRFs, and AChR may be related to the increased activity of distal nerve sprouting, increased activity of AChR, and original NMJ regeneration, thus explaining the better recovery of muscle strength. 

## 8. Conclusion 

Although effort was put forth to create animal models simulating spasticity [114–117], there is currently no universally adopted free-moving animal model that can be used to mimic the spastic changes in clinical situations such as cerebral palsy or stroke [118]. In human clinical situations, a stroke may cause spasticity. In the rat stroke model, such as the suture method or the middle cerebral artery ligation, paralysis instead of spasticity is typically observed over the contralateral side of the brain lesion. Some commonly used spastic animal models, such as spinal cord transection and S2 transection spastic rat tail models, are generated for the purpose of observing neuronal overactivity [118]. Thus, many studies observing the effects of BoNT-A or combined effects of BoNT-A and exercise training in muscles use normal animals [82–85, 87, 94, 96, 109, 110]. To discover a free-moving spastic animal model is therefore an important pursuit for future research. 

**Figure 1:**
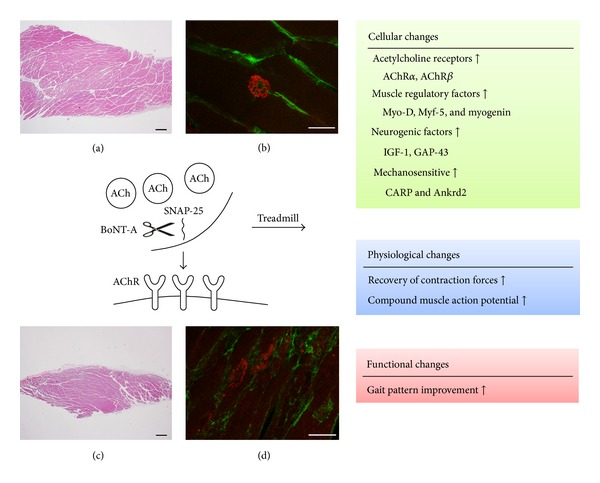
The influence of treadmill exercise on muscle activity and NMJ following BoNT-A injection. Representative pictures of H&E staining of (a) the normal architecture of the gastrocnemius without botulinum toxin injection and (c) atrophy in the gastrocnemius muscle 4 weeks after botulinum toxin injection. Immunohistochemistry staining of neuromuscular junction (NMJ) receptors of (b) the normal configuration of NMJ and (d) changes in the NMJ 4 weeks after BoNT-A injection. A widening of the NMJ (which loses its normal configuration), as well as the diffuse extrajunctional staining of acetylcholine receptors, was noted. Red: NMJ receptors. Green: neurofilament. Scale bar = 50 **μ**m.

The temporal blockade of neuromuscular function by BoNT-A is a useful method for investigating changes in muscle physiology from paralysis to recovery.  Figure 1 summarizes the effects of treadmill training on muscle activity and the NMJ of BoNT-A-induced muscle atrophy. The major effect of BoNT-A is predominantly in the peripheral muscles, especially in the blockade of NMJ functions that cause muscle atrophy and weakness. The adaptation mechanisms induced via treadmill training are multifactorial and include enhanced axon regeneration, activation of the spinal central pattern generator, and functional recovery in the SFI, H-reflex, and CMAP [42, 43, 84, 119]. The molecular mechanisms through which treadmill training promotes synaptic plasticity and functional recovery include the enhancement of IGF-1, MRFs, AChR, and neurotrophin expression [29, 30, 44, 47, 87]. Based on the review, the muscle and nerve recovery effects of treadmill training may counteract the spasticity reduction effect from BoNT-A. When considering the therapeutic strategies of combining BoNT-A and treadmill training in the practice of neurorehabilitation, clinicians should take this potential counteractive effect into consideration. In this review paper, we highlighted the mechanisms of cellular effects following BoNT-A injection and treadmill training and further showed how the combined effects of both treadmill and BoNT-A influence muscle and NMJ activity. This work may improve our understanding of the mechanisms underlying currently used treatments.
